# Preclinical Evaluation of Lipid-Based Nanosystems

**DOI:** 10.3390/pharmaceutics13050708

**Published:** 2021-05-12

**Authors:** Ana Catarina Silva, José Manuel Sousa Lobo

**Affiliations:** 1 UCIBIO/REQUIMTE, MEDTECH, Laboratory of Pharmaceutical Technology, Department of Drug Sciences, Faculty of Pharmacy, University of Porto, 4050-313 Porto, Portugal; slobo@ff.up.pt; 2 FP-ENAS (UFP Energy, Environment and Health Research Unit), CEBIMED (Biomedical Research Centre), Faculty of Health Sciences, University Fernando Pessoa, 4249-004 Porto, Portugal

The use of lipid-based nanosystems, including lipid nanoparticles (solid lipid nanoparticles—SLN, and nanostructured lipid carriers—NLC), nanoemulsions, and liposomes, among others, is widespread. Several researchers have described advantages of the different applications of these nanosystems. For instance, they can increase the targeting and bioavailability of drugs, improving the therapeutic effect. Their use in the cosmetic field is also promising, owing to their moisturizing properties and ability to protect labile cosmetic actives. Thus, it is surprising that only few lipid-based nanosystems have reached the market. This can be explained by the strict regulatory requirements of medicines and the occurrence of unexpected in vivo failure, which highlights the need to conduct more preclinical studies.

Current research is focused on testing the in vitro, ex vivo and in vivo efficacy of lipid-based nanosystems to predict their clinical performance. However, there is a lack of method validation, which compromises the comparison between different studies.

This Special Issue brings together the latest research and reviews that report preclinical studies in vitro, ex vivo and in vivo using lipid-based nanosystems. Readers will find up-to-date information on the most common experiments performed to predict the clinical behavior of lipid-based nanosystems. A series of 15 research articles and a review are presented, with authors from 15 different countries, which demonstrates the universality of the investigations that have been carried out in this area.

F. Fernández-Campos et al. [1] developed lipomers (i.e., lipid core polymeric nanocapsules) loaded with dexamethasone and studied their potential to improve the topical treatment of alopecia areata and other hair-follicle inflammatory diseases. The occurrence of follicular targeting of lipomers suggests that it is possible to have a drug deposition effect within the pilosebaceous unit, which reduces the frequency of administrations, although the safety and efficacy profiles of these lipomers must first be confirmed in clinical trials.

A.B. Nair et al. [2] evaluated the potential of optimized clarithromycin-loaded SLN to improve the ocular permeation of this drug, increasing its therapeutic potential. A 32 full factorial design showed a significant influence of the sonication time and the amount of lipid on the clarithromycin-loaded SLN particle size, entrapment efficiency and drug loading. In addition, the results of the ex vivo permeation studies and in vivo pharmacokinetics studies showed a greater efficacy of the optimized clarithromycin-loaded SLN compared to a drug solution, suggesting that the developed formulation may be a viable drug delivery approach for treating endophthalmitis.

O. Holas et al. [3] investigated the ability of oligonucleotide-loaded self-emulsifying drug delivery systems (SEDDS) to cross intestinal Caco-2 cells. In this study, oligonucleotides were first complexed with cationic lipids to improve lipophilicity and later formulated in SEDDS with negative charge and neutral charge. From the results of their work, the authors suggested the use of SEDDS to improve the oral delivery of oligonucleotides, overcoming difficulties of permeation and stability. However, in vivo testing of oligonucleotide-loaded SEDDS is necessary to obtain more detailed knowledge of this potential for local delivery.

A. Melero et al. [4] developed cyanocobalamin (vitamin B12)-loaded ultraflexible lipid vesicles (liposomes, transfersomes, and ethosomes) with enhanced skin penetration ability, which can be used as a promising alternative to improve the treatment of skin diseases, such as atopic dermatitis and psoriasis. Although vitamin B12 is effective as a nitric oxide scavenger, the hydrophilic character and weight of this molecule limit its diffusion through the skin. In this study, the authors identified the key factors for the efficient production of vitamin B12-loaded lipid vesicles, including size, stability and purification method. In addition, it was observed that the developed lipid vesicles efficiently release vitamin B12 to the deeper layers of the skin, 6 h after the application.

S.A.G. Langeveld et al. [5] studied the use of phospholipid-coated microbubbles as contrast agents for ultrasound molecular imaging and for drug delivery. The authors investigated how lipid handling and phase distribution affect the acoustic behavior of these microbubbles. Varying concentrations of cholesterol were used to modify the lateral molecular packing of 1,2-distearoyl-sn-glycero-3-phosphocholine (DSPC)-based microbubbles, which were produced by a direct method and by an indirect method. The results showed that indirect-produced DSPC microbubbles had a more uniform response to ultrasound than direct-produced DSPC and indirect-produced DSPC-cholesterol microbubbles. Besides, the difference in lipid handling between direct-produced and indirect-produced DSPC microbubbles significantly affected the acoustic behavior, with the latter being the most promising for ultrasound molecular imaging and drug delivery. From these results, the authors concluded that the phase distribution and lipid handling prior to microbubble production significantly affected the acoustic behavior of microbubbles.

M.L. Fanarraga et al. [6] developed doxorubicin-loaded lipid particles (SLPs) (also known as SLN) for the treatment of lung metastatic malignant melanoma. In this study, the authors conducted comparative in vivo experiments in mice, with intravenous doxorubicin-loaded SLPs and intravenous free drug, and observed that the former significantly reduced the number of pulmonary metastatic foci. In addition, prolonged in vitro drug release was observed over 40 days, which suggests the possibility of reaching constant drug levels in situ to inhibit metastatic growth, increasing local drug efficacy, reducing side effects and improving overall survival. Based on these findings, the authors suggested the use of doxorubicin-loaded SLPs as an adjuvant treatment for different types of cancer.

W.Y. Rizg et al. [7] developed fluconazole-loaded sesame oil containing nanotransfersomes to improve the local treatment of oral candidiasis. The Box‒Behnken design was used to evaluate the parameters of the production method that interfere with size, entrapment efficiency, zone of inhibition and ulcer index. The optimized formulation of fluconazole-loaded nanotransfersomes was included in a hyaluronic acid-based hydrogel and characterized to assess its suitability for local application in the oral cavity. Finally, ex vivo and in vivo studies confirmed the superior antifungal efficacy of the developed fluconazole-loaded nanotransfersomes, when compared to fluconazole suspension and to hyaluronic acid hydrogel. The authors suggested that the improved antifungal efficacy of the developed formulation could be related to the synergistic effect of its components, such as hyaluronic acid, sesame oil and fluconazole.

M. Igartua an R.M. Hernandez et al. [8] prepared NLC composed of polyunsaturated fatty acids and evaluated their protective and anti-inflammatory effects aiming to produce a new functional nanocarrier to entrap different therapeutic molecules, acting as a synergistic therapy. In their studies, researchers observed that the developed NLC are biocompatible with both dopaminergic and microglia cells and exhibited neuroprotective effects in dopaminergic neuron cells, after exposition to 6-hydroxydopamine hydrochloride (6-OHDA) neurotoxin, and decreased the proinflammatory cytokine levels in microglia cells, after lipopolysaccharide (LPS) stimuli. From these results, it was concluded that the combination of these new NLC with different therapeutic molecules could be an effective tool for the synergistic treatment of neurogenerative diseases.

R.B. Walker et al. [9] developed and characterized efavirenz-loaded flaxseed oil nanoemulsions. The studies consisted of using a D-optimal design to obtain the surfactant mixture that originates kinetically stable low energy nanoemulsions. The authors proposed the use of flaxseed oil nanoemulsions in oral efavirenz formulations due to the health benefits for patients associated with the use of polyunsaturated fatty acids. In addition, it was also mentioned that flaxseed oil is a renewable inexpensive raw material that can be exploited in the preparation of pharmaceutical dosage forms.

M. Puchkov et al. [10] investigated the in situ formation of liposomes from inert porous calcium carbonate microparticles, called dry powder proliposomal formulations. In the experiments, nifedipine was used as a model of a poorly water-soluble oral drug, and the factors that affect liposome formation and drug encapsulation were studied in different simulated gastrointestinal fluids. From the results, the authors proposed the use of a single-step loading of phospholipids and drugs as a simple and effective method for the production of proliposomal formulations, which is suitable for scale-up.

T. Lajunen et al. [11] developed and characterized light-activated liposomes coated with hyaluronic acid for ocular delivery. In their experiments, the researchers observed that, when compared to liposomes coated with polyethylene glycol (PEG), liposomes coated with hyaluronic acid showed improved plasma stability and greater binding to vitreous humor proteins and collagen-related proteins, which is probably related to the reduced vitreal mobility. From these findings, the authors concluded that the hyaluronic acid-coated light-activated liposomes are a promising alternative for intravenous and ocular drug delivery.

C.A. Prestidge et al. [12] studied the impact of the main characteristics of a nanostructured matrix obtained by silica–lipid hybrids in the construction of optimized solid-state lipid-based formulations for the oral delivery of poorly water-soluble drugs. For the experiments, fenofibrate was used as a drug model and the impact of drug loading, type of lipid and type of silica nanostructure on the in vitro dissolution, solubilization, and solid-state stability of fenofibrate was investigated. Based on their research, the authors proposed that the study of the key characteristics of nanostructured matrix silica–lipid hybrids allows the preparation of tailor-made formulations with regard to the release profile or the desired therapeutic result.

M.P. Scavo et al. [13] evaluated the antitumor effect of immuno-liposomes loaded with 5-fluorouracil and surface-coated with an antibody against the Frizzled 10 protein, which is an overexpressed receptor on the surface of colorectal cancer cells. The experiments were conducted in vitro on two different colorectal cell lines, where the Frizzled 10 protein is overexpressed, called metastatic CoLo-205 and non-metastatic CaCo-2 cells. In addition, several characterization studies were performed on the developed immuno-liposomes and their therapeutic efficacy was evaluated in vitro. The overall results of these studies showed that the cytotoxic activity of 5-fluorouracil increased when it was encapsulated in the immuno-liposomes compared to the free compound. Therefore, the authors suggested the use of these immuno-liposomes for the selective treatment of colorectal cancer, although more studies are needed to investigate the specific mechanisms of cell uptake. Moreover, the potential of these liposomes for inclusion in conventional pharmaceutical dosage forms, such as gastro-resistant capsules or suppositories, was proposed.

K.L. Vine et al. [14] tested the in vitro and in vivo uptake of anti-mitotic N-alkylisatin-loaded liposomes functionalized with plasminogen activator inhibitor type 2 to target the urokinase plasminogen activator and its receptor. Receptor-dependent cytotoxicity and uptake of functionalized liposomes were observed in vitro, which was greater for the plasminogen activator urokinase and its receptor overexpressed in breast cancer cells MDA-MB-231 compared to the low expression of MCF-7 breast cancer cells. The targeting ability of the tested functionalized liposomes was observed in vivo, where there was an increase in accumulation at the site of the primary tumor in an animal model of disease compared to non-functionalized liposomes. Based on these findings, the authors proposed the use of functionalized liposomes to target the plasminogen activator urokinase and its receptor-positive breast cancer cells, improving the treatment of triple-negative breast cancer. In addition, the authors suggested the use of these liposomes to target heterogeneous tumor cells, where the plasminogen activator urokinase and its receptor play a key role in the development of metastases.

A.C. Silva et al. [15] applied the quality by design (QbD) approach to perform a double optimization of rivastigmine-loaded NLC, in which quality target product profile (QTPP) was the requirement of nose-to-brain delivery. The first optimization was related to the selection of critical material attributes (CMAs), including ratios of drug, lipids and surfactants, using a central composite design. Afterwards, the formulation with the best critical quality attributes (CQAs) of particle size, polydispersity index, zeta potential, and encapsulation efficiency was selected for the second optimization, which was related to the production methods (ultrasound technique and high-pressure homogenization). Here, the Box–Behnken design was used to evaluate the same CQAs of the first optimization, with high-pressure homogenization selected as the most suitable production method, although the ultrasound technique has also shown effectiveness. From these results, the authors concluded that QbD is a useful approach for the optimization of NLC formulations with specific requirements.

K. Hart et al. [16] reviewed the literature on the use of liposomes to augment dialysis in preclinical models for both endogenous toxins and intoxicants, in particular, on repurposing the use of liposomes in areas of unmet clinical needs. From their investigations, the authors concluded that the use of pH-gradient liposomes with acidic centers in peritoneal dialysis to increase the extraction of ammonia in hepatic failure is the most studied area, which is under phase 1 of clinical trials. In addition, the use of liposomes to remove exogenous intoxicants and protein-bound uremic and hepatic toxins and the use of liposome-supported enzymatic dialysis have also been studied. In conclusion, the authors believe that the use of liposomes for clinical indications other than drug transport will emerge in the next decade.

We would like to thank all the authors of this Special Issue for contributing with high quality works. We also acknowledge all the reviewers who critically evaluated the articles. In addition, we would like to thank the Assistant Editor, Ms. Claudia Li, for her kind help.
